# Risk of Stillbirth in the Relation to Water Disinfection By-Products: A Population-Based Case-Control Study in Taiwan

**DOI:** 10.1371/journal.pone.0033949

**Published:** 2012-03-23

**Authors:** Bing-Fang Hwang, Jouni J. K. Jaakkola

**Affiliations:** 1 Department and Graduate Institute of Occupational Safety and Health, College of Public Health, China Medical University, Taichung, Taiwan, Republic of China; 2 Center for Environmental and Respiratory Health Research, Institute of Health Sciences, University of Oulu, Oulu, Finland; Medical Faculty, Otto-von-Guericke University Magdeburg, Medical Faculty, Germany

## Abstract

**Background:**

Few epidemiological studies that have assessed the relation between water disinfection by-products (DBPs) and the risk of stillbirth provide inconsistent results. The objective was to assess the relation between exposure to water disinfection by-products and the risk of stillbirth.

**Methods:**

We conducted a population-based case-control study of 3,289 cases of stillbirth and a random sample of 32,890 control subjects from 396,049 Taiwanese newborns in 2001–2003 using information from the Birth Registry and Waterworks Registry in Taiwan. We compared the risk of stillbirth in four disinfection by-product exposure categories based on the levels of total trihalomethanes (TTHMs) representing high (TTHMs 20+ µg/L), medium (TTHMs 10–19 µg/L), low exposure (TTHMs 5–9 µg/L), and 0–4 µg/L as the reference category. In addition, we conducted a meta-analysis of the results from the present and 5 previous studies focusing on stillbirth.

**Findings:**

In logistic regression analysis adjusting for gender, maternal age, plurality, conception of season and population density of the municipality where the mother lived during pregnancy, the odds ratio (OR) for stillbirth was 1.10 (95% CI 1.00–1.21) for medium exposure and 1.06 (95% 0.96–1.17) for high exposure compared to reference category. In the meta-analysis, the summary odds ratio for stillbirth (1.11, 95% CI: 1.03, 1.19) was consistently elevated.

**Conclusions:**

The present study is consistent with the hypothesis that the risk of stillbirth is related to prenatal exposure to disinfection by-products. This finding on stillbirth is consistent with previous epidemiologic studies, which strengthens the weight of evidence.

## Introduction

Water chlorination is a widely used and efficient method to reduce the occurrence of water-borne diseases, and has been one of the most successful public health measures introduced in the 20^th^ century. In early 1970s, some volatile halogenated organic compounds, such as chloroform, were identified in chlorinated surface waters containing high levels of natural organic material [1]. Later many other disinfection by-products, such as other trihalomethanes (THMs), haloacetic acids, chlorophenols, chloral hydrate, and haloacetonitriles have been identified, most from the process of chlorination, but also from chloramination, chlorine dioxide disinfection, and ozonation [2]. Generally, the THMs, including chloroform, bromodicholormethane, dibromochlormethane, and bromoform are the most prevalent in chlorinated surface water [3]. They are routinely measured throughout water works in Taiwan.

Six previous epidemiological studies have assessed the relation between exposure to water disinfection by-products (DBPs) and the risk of stillbirth; they have provided inconsistent results [4]–[9]. Two of the studies indicated no significant association between DBPs and stillbirth [4]–[5]. Two other authors, both using the same dataset from Nova Scotia, reported that the risk of stillbirth was related to exposure to high level of TTHM (>100 ug/l) [6]–[7]. Later, a case-control study in Nova Scotia found that TTHMs level of 80 or more ug/l increased the risk of stillbirth compared with no exposure to TTHMs, yielding an adjusted odds ratio (OR) of 2.2 (95% CI: 1.1–4.4) [8]. A study conducted in three water regions in England indicated an increased risk of stillbirth in an area with high level of TTHM (≥60 ug/l) (OR = 1.11, 95% CI: 1.00–1.23) [9].

We conducted a population-based case-control study of 3,289 cases of stillbirth and a random sample of 32,890 control subjects from 396,049 Taiwanese newborns in 2001–2003 to assess the effect of water disinfection by-products on the stillbirth. We also synthesized quantitatively our results with previous epidemiologic studies [4]–[9], which focused on stillbirth.

## Methods

### Study design

We conducted a population-based case-control study of stillbirth. The source population comprised of all 721,289 births registered by the Taiwanese Birth Registry from January 1, 2001 to December 31, 2003. We focused on five water regions, which were served by only one type of water treatment plant. The final study population included 396,049 infants. We identified all the cases of stillbirth without any birth defects in the source population during the study period. Birth records in the registry were sorted by the date of birth. Control subjects were selected randomly from the source population. The study protocol was approved by the Institutional Review Board of China Medical University, and it complied with the principles outlined in the Helsinki Declaration [10]. The reason for lack of informed consent was that the data were analyzed anonymously.

### Definition and selection of cases

All births delivered within 15 days are compulsorily reported to the Taiwan Birth Registration. Taiwanese pregnant women are 99% covered by national health insurance and access to prenatal care is free of charge and there are at least 10 visits during pregnancy. The follow-up time is from the 1^st^ month after conception through 7 days after birth. Stillbirths are mostly diagnosed by physician, most often by pediatrician. A validation study of the Taiwanese birth registration reported a low percentage of missing information (1.6%) and good validity (sensitivity and specificity was 92.8%, and 99.6% respectively) and reliability (Cohen's k statistics was 0.92) for stillbirth [11].

We identified all stillbirths without any birth defects (ICD-9-CM: 740–758) from the Taiwanese Birth Registry from 2001 to 2003. The fetal death was defined as a stillbirth after 20 weeks of gestation. We included fetuses over 20 weeks of gestational age without maternal smoking during pregnancy (15 cases (0.3%) excluded due to maternal smoking). A total of 3,289 subjects of non-smoking mothers were identified with sufficient information on gestational age (>20 weeks)

### Selection of control subjects

The control subjects were randomly selected from the source population without any birth defects and maternal smoking during pregnancy (340 controls (0.05%) excluded due to maternal smoking). The eligibility criteria included: born during the study period; no birth defects present; and sufficient information on gestational age and TTHMs. The case-control ratio was approximately 1∶10 to meet optimal statistical power. The final study population included 32,890 controls.

### Exposure assessment

Assessment of exposure was based on municipal-level water quality information on concentrations of total trihalomethanes (TTHMs), and the mother's place of residence during pregnancy. One or more waterworks serve each municipality and the water treatment plants seldom serve across the municipality borders. The Taiwanese water supply system is quite simple. Two hundred water treatment plants from Taiwanese Water Supply Corporation (TWSC) serving about 21 million people (90%) chlorinate their water, and privately owned wells (groundwater) serving about 2 million people (10%) do not use chlorination.

The general hypothesis was that exposure to disinfection by-products through tap water increases the risk of stillbirth. We divided water treatment plants according to the levels of total trihalomethanes (TTHMs) (in µg/L) as a quantitative measure of the water disinfection by-products. The TTHM level is recorded routinely in most of the water treatment plants. Under the regulations operating during the study period, the standard sampling frequency for TTHMs was a minimum of four samples per year for each water treatment plant. We assessed exposure by calculating a weighted average of the modeled quarterly TTHM estimates for the appropriate water treatment plants during the date of conception and the date of birth. The weighting was based on the proportion of the trimester falling into each quarterly period. The number of measurements and distribution of TTHMs between three exposure categories and reference category are shown in Table 1.

**Table 1:** The number of measurements and distribution of TTHMs between three exposure categories and reference category.

Exposure categories	No of samples	Mean±SD (µg/L)	Minimum (µg/L)	25 percentile (µg/L)	Median (µg/L)	75 percentile (µg/L)	Maximum (µg/L)
Reference (TTHMs 0–4 µg/L)	528	3.64±0.95	0.85	3.50	3.80	4.06	4.96
Low (TTHMs 5–9 µg/L)	168	5.57±0.96	5.00	6.25	7.15	8.05	9.50
Medium (TTHMs 10–19 µg/L)	240	16.48±2.94	10.68	14.95	15.57	17.57	19.53
High (TTHMs 20+ µg/L)	228	23.24±2.27	20.35	21.90	22.70	24.90	32.65

### Covariates

We used routine birth registry data to construct the following covariates: gender of infant (male; female), maternal age (<20 years; 20–34 years; > = 35 years), plurality (singleton; and multiple birth), and maternal health status defined as the presence of any of the following diseases or conditions: diabetes mellitus, anemia (HCT<0.30/HGB<0.10), cardiac disease, acute or chronic lung disease, genital herpes, hydraminios/oligohydramnios, chronic hypertension, pregnancy-associated hypertension, eclampsia, imcompetent cervix, renal disease, Rh sensitization, uterine bleeding (yes; no). We received municipal level data from the Department of Household Registration Affairs, Taiwanese Population Data services, which were used to construct municipal level population density, which is a measure of the proportion of urban population in the municipality.

### Statistical methods

We estimated the prevalence (%) of the stillbirth with 95% confidence intervals based on binomial distribution. We compared the risk of stillbirth in three exposure categories (TTHMs 20+ µg/L; TTHMs 10–19 µg/L; TTHMs 5–9 µg/L) to the risk in the reference category with the lowest concentrations of TTHMs (0–4 µg/L). We used prevalence odds ratio as a measure of association and we applied logistic regression to estimate the adjusted odds ratios. The goodness of fit was tested with likelihood ratio tests (LR) to assess whether or not a variable contributes significantly to the model. First, we fitted a full model with a complete set of covariates. To elaborate sources of confounding, we fitted models with different combinations of covariates and compared the effect estimates from models with and without the covariate of interest. If the adjusted results differed from unadjusted results by >10%, the variable was included in the model. To evaluate the effect modification, we systematically compared effect estimates on different levels of covariates. We tested the trend in the exposure-outcome relations in logistic regression by fitting an ordinal scale exposure variable (0, 1, 2, and 3) based on the exposure categories.

We searched the Medline data base from 1966 through March 2011, using the following key words: (water disinfection OR trihalomethanes OR disinfection by-products) AND (stillbirth). In addition, we searched primary references from the identified publications and reviewed manually issues of the Archives of Environmental Health, Environmental Health Perspectives, Environmental Research, and Epidemiology. We considered all epidemiological studies that assessed the relation between exposures to chlorination disinfection by-products, either directly or indirectly, and stillbirth.

Two authors independently reviewed the articles, extracted data, and assessed the validity of the studies. We applied the following inclusion criteria on the basis of the type of study, study population, exposure definition, and outcome definition. We accepted *a priori* all studies with individual as the unit of observation, including cross-sectional, cohort and case-control studies.

Finally, we conducted a meta-analysis of the present and the other available studies of stillbirth in relation to water chlorination [4], [5], [8], [9]. The numbers of studies screened, assessed for eligibility, and included in the review, with reasons for exclusions at each stage, ideally with a flow diagram are presented in the Figure S1. We calculated summary odds ratios using both the fixed-effects and random-effects models. The fixed-effects model was calculated using the Mantel-Haenszel method with inverse variances of individual effect estimates as weights [12]. The random-effects models were calculated using the method of DerSimonian and Laird [13]. We calculated summary odds ratios using the estimates from the contrast between the highest and the reference category. We studied heterogeneity of the study-specific effect estimates by plotting the measures of effect and applying Q statistic. We elaborated the heterogeneity between the specific effect estimates, but presented systematically summary estimates from both fixed and random-effects models to offer readers a possibility for their own informed judgment. In case of heterogeneity between study-specific effect estimates, we applied stratified analysis to explain the heterogeneity by the aforementioned study characteristics. We also performed sensitivity analysis with and without the study with the largest sample size to identify the impact of this individual study on the results.

## Results

Among 369,049 newborns in the study population, we identified 3,289 stillbirths (0.89%). Table 2 displays the study population according to the exposure categories. The municipalities in the high exposure category had a lower population density compared with the reference municipalities. A larger proportion of cases than controls were male, had older mother, were multiple birth and area of higher population density (Table 3). We adjusted for these factors in the multivariate analysis.

**Table 2:** Characteristics of the study population (N = 396,049) according to the categories of exposure, Taiwan, 2001–2003.

Characteristic	Subcategory	TTHMs (0–4 µg/L) Reference N (%)	TTHMs (5–9 µg/L) Low N (%)	TTHMs (10–19 µg/L) Medium N (%)	TTHMs (20+ µg/L) High N (%)	Total N (%)
Gender of infant	Male	95,027 (52.2%)	29,128 (52.1%)	43,155 (52.1%)	39,413 (52.3%)	206,723(52.2%)
	Female	86,958 (47.8%)	26,822 (47.9%)	39,642 (47.9%)	35,904 (47.7%)	189,326 (47.8%)
Maternal age	<20 years	6,419 (3.5%)	1,838 (3.3%)	3,499 (4.2%)	3,461 (4.6%)	15,217 (3.8%)
	20–34	156,087 (85.8%)	46,871 (83.8%)	72,402 (87.4%)	65,947 (87.6%)	341,307 (86.2%)
	35-	19,476 (10.7%)	7,241 (12.9%)	6,893 (8.3%)	5,909 (7.8%)	39,519 (10.0%)
Maternal diabetes mellitus	Yes	418 (0.2%)	189 (0.3%)	179 (0.2%)	144 (0.2%)	930 (0.2%)
	No	181,567 (99.8%)	55,761 (99.7%)	82,618 (99.8%)	75,173 (99.8%)	395,119 (99.8%)
Plurality	Singleton	176,791 (97.1%)	54,383 (97.2%)	80,755 (97.5%)	73,442 (97.5%)	385,371 (97.3%)
	Multiple birth	5,194 (2.9%)	1,567 (2.8%)	2,042 (2.5%)	1,875 (2.5%)	10,678 (2.7%)
Population density (no of people/km^2^)*	<1000	29,881 (16.4%)	6,697 (12.0%)	20,387 (24.6%)	26,392 (35.2%)	83,357 (21.1%)
	1000–5000	71,005 (39.0%)	27,257 (48.7%)	23,569 (28.5%)	31,823 (42.5%)	153,654 (38.8%)
	5000	81,099 (44.6%)	21,996 (39.3%)	38,841 (46.9%)	16,738 (22.3%)	158,674 (40.1%)
Total		181,985 (100%)	55,950 (100%)	82,797 (100%)	75,317 (100%)	396,049 (100%)

**Table 3:** Distribution of demographic variables among case and controls.

Characteristics	Subcategory	Cases N (%)	Controls N (%)
Gender of infant		χ^2^ = 3.34; p = 0.06	
	Male	1755 (53.4)	17001 (51.7)
	Female	1534 (46.6)	15889 (48.3)
Maternal age		χ^2^ = 309; p<0.001	
	<20 years	194 (5.9)	1247 (3.8)
	20–34	2474 (75.2)	28426 (86.4)
	35-	620 (18.9)	3215 (9.8)
Plurality		χ^2^ = 336; p<0.001	
	Singleton	3002 (91.3)	31986 (97.3)
	Multiple birth	287 (8.7)	904 (2.7)
Population density (no of people/km^2^)*		χ^2^ = 401; p<0.001	
	<1000	619 (18.8)	6857 (20.9)
	1000–5000	1210 (36.8)	12742 (38.8)
	>5000	1457 (44.3)	13262 (40.4)
Total		3289 (100)	32890 (100)

Table 4 provides evidence that the risk of stillbirth is related to medium (adjusted OR 1.10, 95% CI: 1.00, 1.21) and high exposure (adjusted OR 1.06, 95% CI: 0.96, 1.17) compared to reference category, but there is no clear dose-response pattern.

**Table 4:** Odds ratios of stillbirth according to exposure to TTHMs in Taiwan 2001–2003.

Stillbirth	TTHM (0–4 µg/L) Reference	TTHM (5–9 µg/L) Low	TTHM (10–19 µg/L) Medium	TTHM (20+ µg/L) High
Cases (%)	1492 (45.4)	480 (14.6)	705 (21.4)	612 (18.6)
Controls (%)	15127 (46.0)	4732 (14.4)	6730 (20.5)	6301 (19.2)
OR (95% CI)	1.00	1.03 (0.92–1.15)	1.06 (0.97–1.17)	0.99 (0.89–1.09)
aOR (95% CI)	1.00	1.02 (0.92–1.14)	1.10 (1.00–1.21)	1.06 (0.96–1.17)

cOR: crude odds ratio; aOR: adjusted odds ratio. Logistic regression analysis adjusting for maternal age, plurality, and population density.

Table 5 shows the characteristics of the present study and the five previous studies and gives the study-specific adjusted odds ratios for the available outcomes. Exposure assessment constituted the main source of differences between the studies. We classified the studies according to the main approaches, A) water chlorination to chloramination [4], and B) routine measurements of trihalomethanes [5]–[9]. These categories were applied in the stratified analyses when the individual studies were heterogeneous.

**Table 5:** Summary of the results from previous and present studies.

Authors	Aschengrau1993	Bove1995	Dodds 1999	Toledano 2005	Dodds 2004	Hwang2011
Location	Massachusetts	New Jersey	Nova Scotia	England	Nova Scotia	Taiwan
Type of study	Case-control, hospital-based	Cross-sectional, population-based	Retrospective cohort study	Cross-sectional, population-based	Population-based case-control	Population-based case-control
Study Population	1,490 controls	80,938 births	13,728	920,571	398 controls	32890 controls
No of stillbirth	121	594	197	60,641	112	3,289
Exposure	Chlorined surface water	TTHMs >100 ppb	TTHMs > = 100 µg/l	TTHMs > = 60 µg/l	TTHMs > = 80 µg/l	TTHMs > = 20 µg/l
Reference	Chloraminated surfacce water	TTHMs < = 20 ppb	TTHMs 0–49 µg/l	TTHMs <30 µg/l	TTHMs 0 µg/l	TTHMs 0–4 µg/l
Risk Ratio	2.6 (0.9–7.5)	<1.0 (no CI given)	1.66 (1.09–2.52)	1.11 (1.00–1.23)	2.2 (1.1–4.4)	1.06 (0.96–1.17)

**Figure 1:**
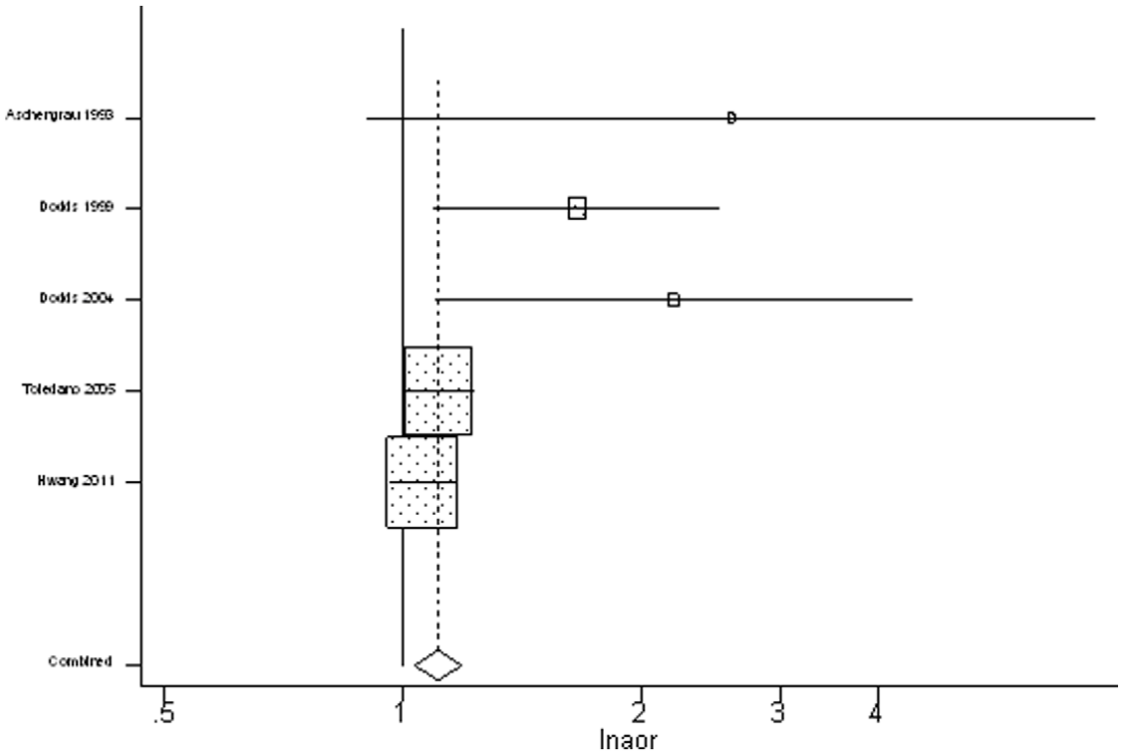
Tree Plots of Study-specific and Summary Effect Estimates for all available studies.

As shown in table 6, the summary odds ratios for stillbirth (random-effects model: summary OR 1.11, 95% CI: 1.03, 1.19, heterogeneity: p = 0.031) provided consistent evidence of an increased risk, but showed some heterogeneity. Figure 1 shows tree plots of the study-specific and summary-effect estimates of stillbirth for all available studies.. The area of the plot indicates the relative amount of information and a horizontal line through the plot signifies the 95% confidence interval. The sensitivity analysis for stillbirth revealed that the English and Taiwanese studies were responsible for the heterogeneity. After exclusion of both studies, the summary odds ratio was homogeneous and substantially elevated (fixed-effects model: 1.79 (95% CI: 1.25, 2.56, heterogeneity: p = 0.495).

**Table 6:** A comparison of the results from the present, Massachusetts, and Nova Scotia.

Stillbirth	Study area	Fixed-Effects Model Summary OR (95% CI)	Random-Effects Model Summary OR (95% CI)	Q-statistic/P-value*
All	Massachusetts 1993 Nova Scotia1999 Nova Scotia 2004 England 2005 Taiwan 2011	1.11 (1.03–1.19)	1.21 (1.02–1.43)	10.63/0.031
Stratum A	Massachusetts 1993	2.6 (0.9–7.5)		
Stratum B	Nova Scotia 1999 Nova Scotia 2004 England 2005 Taiwan 2011	1.11 (1.03–1.18)	1.17 (1.01–1.37)	8.13/0.043
Stratum C	Massachusetts 1993 Nova Scotia 1999 Nova Scotia 2004 England 2005	1.16 (1.05–1.28)	1.55 (1.04–2.31)	9.044/0.029
Stratum D	Massachusetts 1993 Nova Scotia 1999 Nova Scotia 2004 Taiwan 2011	1.11 (1.01–1.22)	1.55 (1.01–2.39)	10.63/0.014
Stratum E	Nova Scotia 1999 Nova Scotia 2004 England 2005	1.15 (1.04–1.27)	1.45 (0.97–2.16)	6.79/0.034
Stratum F	Nova Scotia 1999 Nova Scotia 2004Taiwan 2011	1.10 (1.00–1.21)	1.44 (0.93–2.23)	8.12/0.017
Stratum G	Nova Scotia 1999 Nova Scotia 2004	1.79 (1.25–2.56)	1.78 (1.24–2.58)	0.465/0.495
Stratum H	England 2005 Taiwan 2011	1.08 (1.01–1.22)	1.09 (1.00–1.24)	0.402/0.526

* P<0.05 indicates that a random-effects model is more appropriate.

Startum A = chlorinated and chloramined surface water; Stratum B = routine measurement of trihalomethanes; Startum C = excluded Taiwan study; Startum D = excluded England study; Startum E = focusing on routine measurement of trihalomethanes and excluded Taiwan study; Startum F = focusing on routine measurement of trihalomethanes and excluded England study; G = focusing on routine measurement of trihalomethanes and excluded both England and Taiwan studies; H = included England and Taiwan Studies.

## Discussion

Assessment of potential effects of exposure to disinfection by-products on the risk of stillbirth is problematic because of diversity of the exposure assessment. Few previous studies [4]–[9] have focused on stillbirth and five have assessed the associations between exposure to TTHMs and the risk of stillbirth [5]–[9].

Our results showed a consistent association between exposure to TTHMs and the risk of stillbirth. The effect estimates for stillbirth were substantially elevated in the medium and high exposure compared to the reference category. The meta-analysis of stillbirth together with the Massachusetts, Nova Scotian, and English data strengthened the evidence for stillbirth.

### Validity of results

The present study had enough power to estimate the relations between exposure to disinfection by-products and the risk of stillbirth, which could be relevant adverse effects based on previous literature. The meta-analysis together with the Massachusetts and Nova Scotia results improved the precision of the finding on stillbirth. We excluded half of the births because of insufficient water quality data. This exclusion was unlikely to introduce selection bias, because it was made on municipality level and the characteristic of the excluded individuals did not differ substantially from the included (χ^2^ test; p>0.05), as shown in Table S1. Because there is a possibility that the presence of birth defects may augur other exposure that also may mediate the risk of stillbirth, we excluded all case and control subjects with any birth defect.

Our outcome assessment was based on birth registration, as in the vast majority of the previous studies of disinfection by-products and stillbirth [5]–[7], rather than clinical examination for the purposes of the study. This is a source of misclassification, which is likely to be random, i.e. not related to the exposure of interest, and thus lead to underestimation of the effect estimates. The sources of misclassification could include variation in diagnostic criteria and errors in reporting information provided by physician or hospital. Important features in the Taiwan national health care system limit the amount of outcome misclassification. Taiwanese pregnant women are almost all covered by health insurance (>99%,) and access to prenatal care is free and good (at least 10 visits during pregnancy). The clinical surveillance of pregnancy begins at 1 month after conception and continues through 7 days after birth. Stillbirth was mostly diagnosed by physician, most often by paediatrician. In general, the stillbirth might be underreported, because we did not include gestational age less than 20 weeks, and induced abortions due to stillbirth. This underreporting was likely to be non-differential, i.e. not related to exposure. In most situations non-differential misclassification of a binary outcome will produce bias toward the null, provided that the misclassification is independent of other errors [14].

A major challenge of this study was the imprecision of exposure assessment from using aggregate municipal measures for classifying individual exposures. We had no information on the amounts or sources of water consumed by pregnant women and exposure to volatile disinfection by-products through inhalation and dermal absorption, which might decrease the accuracy of exposure assessment. In addition we did not completely collect or integrate information on exposure through different routes and the accuracy of exposure assessment was likely to be affected by women's uncertainty of recalling their water-use behaviors. Future exposure assessment should include exposure through multiple routes such as bathing, showering and swimming, as well as water consumption from home and work. Biomarkers can be useful to estimate the uptake of the chlorination by-products and integrate multiple exposure routes. Although exhaled breath has been used to estimate chloroform uptake, the short biological half-life (approximately 30 min) is substantial limitation [3]. Dichloroacetic acid (DCAA) and trichloracetic acid (TCAA) are two major nonvolatile disinfection by-products produced as a result of chlorination of drinking water. Urine TCAA appears to be a valid biomarker of ingestion exposure to TCAA from chlorinated water, but the half-life of this marker is only 48 hours [15].

Unfortunately, we do not have sufficient information on vitamin consumption, medication, and genetic factors. Adjustment for population density adjusted indirectly for municipal differences in these behavior factors, but also eliminated partly regional differences in TTHM levels between rural and urban. Residential mobility during pregnancy may be an issue in our study. Two studies conducted in the United States showed that 25% [16] and 37% [17] of women moved during pregnancy. We have no information on the change of residence during pregnancy in Taiwan birth registration. Since the misclassification of exposure is likely to be non-differential related to the outcomes of interest, the level of such misclassification would likely bias the effect estimations toward to the null. We systematically carried out stratified analyses in different categories of exposure and other covariates to elaborate the residual confounding and potential effect modification. The stratified analyses did not indicate any major confounding or effect modification.

The interpretation of meta-analysis is more difficult when the specific effect estimates differ substantially from each other, especially if there are estimates both below and above unity. The random-effects model has become a standard approach to incorporate heterogeneity. We elaborated the heterogeneity between the specific effect estimates, but presented systematically summary estimates from both fixed and random-effects models to offer readers a possibility for their own informed judgment. We also made an attempt to explain the observed heterogeneity, although the small number of studies limited the applicability of this approach. The type of study, study populations, and outcome assessment were relatively similar between the summary studies, but there were different approaches to exposure assessment. The present and three previous studies using routine measurements of TTHMs [6]–[8] constituted a rather homogeneous group, but different exposure contrasting. Also the Massachusetts study used water source and chlorination practice as the basis for exposure assessment [4]. Additionally, the sensitivity analysis indicated that the present study had a substantial impact on the summary effect.

### Publication bias

**Figure 2:**
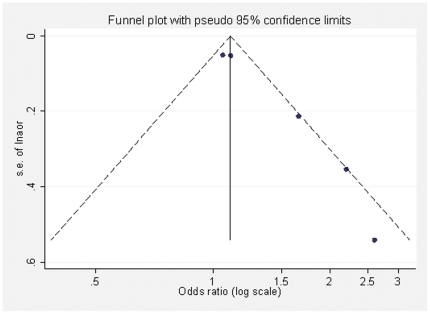
Funnel plot of the effect estimates (ln OR) by their SEs (s.e. of ln OR). The vertical line indicates the summary effect estimate (1.11) from the fixed-effects model according to the SE, and the dashed lines showpseudo 95% confidence limits for the summary effect estimate.

We assessed the potential for publication bias by using a funnel plot, as shown in Figure 2. The vertical line indicates the summary effect estimate from the fixed-effects model (1.11) and the corresponding pseudo 95% confidence limits converging as a function of the SE of the effect estimate. The larger studies with small SEs of ln OR seem to be scattered symmetrically around the summary effect estimate, whereas the funnel plot shows substantial heterogeneity among the smaller studies with large SEs, with an imbalance toward large positive effect estimates. The pattern is consistent with a typical publication bias, in which the effect estimates from small studies would be biased toward large positive values. Although the funnel plots has shown evidence for publication bias, funnel-plot asymmetry may reflect other types of bias or even result from the true effect differing between small and large studies. It should, thus, be seen as displaying the evidence for “small study effects” in general rather than publication bias in particular [18].

### Synthesis with previous knowledge

Four [6]–[9] of the six previous studies [4]–[9] provided evidence of an increase in the risk of stillbirth related to exposure to disinfection by-products. A hospital-based case-control study from Massachusetts contrasting chlorination to chloramination reported an effect estimate which was elevated, but not statistically significant [4]. One population-based cross-sectional, two retrospective cohort and one population-based case-control studies focusing on stillbirth provide a clear pattern of the relation of exposure to TTHMs [6]–[9]. A population-based case-control study in New Jersey reported no significant association between exposure to TTHMs and the risk of stillbirth [5]. In the present study the risk of stillbirth was elevated in the medium and high exposure categories. The meta-analysis of the five available studies provided a conclusive, but heterogeneous summary estimate.

Our study suggests that prenatal exposure to disinfection by-products increases the risk of stillbirth at much lower levels than found in United States [4], Canadian [6]–[8], and England [9] drinking water sources, probably explained by qualitative geographic differences in the levels of natural organic matter (disinfection by-products precursor) or higher concentration of other non-volatile disinfection by-products (e.g. haloacetic acids). The present and the four previous studies from Massachusetts, Nova Scotia, and England suggest also an increased risk of stillbirth.

### Biologic mechanisms

The specific mechanisms for the effects of trihalomethanes (THMs) on the stillbirth are still unknown. Some animal studies show reproductive and developmental toxicity of some of these compounds, such as chloroform (CF) and bromodichloromethanes (BDCM), when given at high doses [3]. There is evidence that metabolites of chloroform may accumulate in the amniotic fluid of pregnant mice [19]. In addition, BDCM can disrupt syncytiotrophoblast formation and inhibit chorionic gonadotrophin secretion in vitro [20]. This implies that the placenta is a likely target of BDCM toxicity in human and thus BDCM may have fatal effects on fetus.

An alternative explanation is that THMs may lead to stillbirth via genetic damage to maternal gametes. For example, CF may be oxidatively metabolized and decomposed to electrophilic phosgene, which is more likely to react and bind to cell components including proteins, phospholipid polar heads, and reduced glutathione [21]. This may result in chromosomal abnormalities, enzymatic malfunction, and disruption of cellular membranes, all of which could interfere with uterine development or directly influence on the conceptus.

Although THMs are the most prevalent in chlorinated water, they are also markers of a complex mixture of disinfections by-products. Some animal studies also show reproductive and developmental toxicity of haloacetic acid, non-volatile disinfection by-products, such as dichloroacetic acid (DCAA) and trichloracetic acid (TCAA). Further detailed toxicological assessments of mixtures of chlorination by-products are also needed, as humans are most commonly exposed to complex mixtures of these compounds rather than to a single compound [22].

### Summary

The present study suggests that prenatal exposure to disinfection by-products increases the risk of stillbirth. The finding on stillbirth is consistent with previous epidemiologic studies, which strengthens the weight of evidence.

## Supporting Information

Figure S1 **PRISMA 2009 Flow Diagram.**
(DOC)

Table S1 **Characteristics of included population, excluded population, and total population in Taiwan 2001–2003.**
(DOC)
