# Supplementary material for: Risk of Stillbirth in the Relation to Water Disinfection By-Products: A Population-Based Case-Control Study in Taiwan
Source: PLoS One. 2012 Mar 23;7(3):e33949. doi: 10.1371/journal.pone.0033949 (PMC3311556; doi:10.1371/journal.pone.0033949)
Supplement: Figure S1 — PRISMA 2009 Flow Diagram. (DOC) [file pone.0033949.s001.doc]

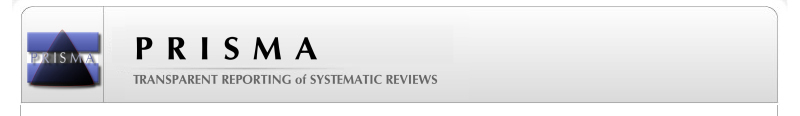
**PRISMA 2009 Flow Diagram (Supplement 1)**

**Screening**

**Included**

**Eligibility**

**Identification**

Records identified through database searching
(n = 9 )

Additional records identified through other sources
(n = 1 )

Records after duplicates removed
(n = 0 )

Records screened
(n =10 )

Records excluded
(n = 0 )

Full-text articles assessed for eligibility
(n = 10 )

Full-text articles excluded, with review articles, without assessing stillbirth or no confidence interval was given (n =5 ).

Studies included in qualitative synthesis
(n = 5 )

Studies included in quantitative synthesis (meta-analysis)
(n = 5 )
