# Supplementary material for: Risk of Stillbirth in the Relation to Water Disinfection By-Products: A Population-Based Case-Control Study in Taiwan
Source: PLoS One. 2012 Mar 23;7(3):e33949. doi: 10.1371/journal.pone.0033949 (PMC3311556; doi:10.1371/journal.pone.0033949)
Supplement: Table S1 — Characteristics of included population, excluded population, and total population in Taiwan 2001–2003. (DOC) [file pone.0033949.s002.doc]

**Table S1. Characteristics of included population, excluded population, and total population in Taiwan 2001-2003.**

|  | **Included population** | | **Excluded population** | | **Total population** | |
| --- | --- | --- | --- | --- | --- | --- |
| Characteristics | N | % | N | % | N | % |
| Total | 396,049 | 100 | 325,240 | 100 | 721,289 | 100 |
| Gender of infant |  | | |  |  |  |
| Male | 206,723 | 52.2 | 170,385 | 52.4 | 377,108 | 52.3 |
| Female | 189,326 | 47.8 | 154,524 | 47.6 | 343,850 | 47.7 |
| Maternal age |  | | |  |  |  |
| <20 years | 15,217 | 3.8 | 16,223 | 5.0 | 31,440 | 4.4 |
| 20-34 | 341,307 | 86.2 | 282,381 | 86.8 | 623,688 | 86.5 |
| 35- | 39,519 | 10.0 | 26,631 | 8.2 | 66,150 | 9.2 |
| Maternal diabetes mellitus |  | | |  |  |  |
| Yes | 930 | 0.2 | 876 | 0.3 | 1,806 | 0.3 |
| No | 395,119 | 99.8 | 324,364 | 99.7 | 719,483 | 99.7 |
| Plurality |  | | |  |  |  |
| Singleton | 385,371 | 97.3 | 316,903 | 97.4 | 702,274 | 97.4 |
| Multiple birth | 10,678 | 2.7 | 8,337 | 2.6 | 19,015 | 2.6 |
| Population density  (no of people/km^2^)* |  | | |  |  |  |
| <1000 | 83,357 | 21.1 | 94,304 | 29.5 | 177,661 | 24.8 |
| 1000-5000 | 153,654 | 38.8 | 144,069 | 45.0 | 297,723 | 41.6 |
| >5000 | 158,674 | 40.1 | 81,817 | 25.6 | 240,491 | 33.6 |
